# Technological Innovation in Outpatient Assistance for Chronic Liver Disease and Liver Transplant Patients During the Coronavirus Disease Outbreak: A Method to Minimize Transmission

**DOI:** 10.6061/clinics/2020/e1946

**Published:** 2020-05-18

**Authors:** Suzane Kioko Ono, Wellington Andraus, Debora Raquel Benedita Terrabuio, Vilson Cobello-Júnior, Lilian Arai, Liliana Ducatti, Luciana Bertocco de Paiva Haddad, Luiz Augusto Carneiro D’Albuquerque, Flair José Carrilho

**Affiliations:** I Divisao de Gastroenterologia e Hepatologia Clinica, Departamento de Gastroenterologia, Hospital das Clinicas HCFMUSP, Faculdade de Medicina, Universidade de Sao Paulo, Sao Paulo, SP, BR.; II Divisao de Transplante de Orgaos Digestivos, Departamento de Gastroenterologia, Hospital das Clinicas HCFMUSP, Faculdade de Medicina, Universidade de Sao Paulo, Sao Paulo, SP, BR.; III Nucleo Especializado em Tecnologia da Informacao NETI, Hospital das Clinicas HCFMUSP, Faculdade de Medicina, Universidade de Sao Paulo, Sao Paulo, SP, BR.; IV Hackmed.

The rapid spread of the coronavirus pandemic worldwide warrants a huge transformation in the health assistance system and patient care. Most countries affected by coronavirus disease (COVID-19) are in lockdown, with restrictions on the movement of people and in services to prevent rapid transmission that may consequently lead to a health system collapse. The disease is known to cause severe acute respiratory syndrome (SARS), especially in the elderly population and in patients with comorbidities.

Patients with chronic liver diseases can progress to liver cirrhosis and develop complications, such as ascites, pleural effusion, renal dysfunction, diabetes, and obesity (non-alcoholic fatty liver disease). Some of them who have undergone liver transplantation are in an immunosuppressed state and thus are prone to infections. Currently, we do not have substantial data on the effects of SARS coronavirus-2 in chronic liver disease and liver transplant patients, who are known to be at high risk for opportunistic infections. These patients have a severe illness or have recently been operated upon and need to attend their outpatient appointments (1,2).

Protection of health care workers is another major concern in this scenario, as several patients with respiratory symptoms of COVID-19 visit clinics, and clinic secretaries, nurses, attenders, assistants, and doctors work directly with the patients. Furthermore, the world is facing a huge shortage of health care workers and specialized professionals, who are very difficult to replace during this pandemic. The WHO and Pan American Health Organization are emphasizing on the need for protection of health care workers: “Dr. Etienne highlighted that countries must also protect their health personnel as never before” (3).

In this context, we have started using robotic assistance for first contact with patients who arrive at our clinic. A nurse controls the robot remotely from a station, from where she can talk to and give directions and orders to patients and, most importantly, screen symptomatic patients with respiratory illnesses and guide them directly to specific rooms in which they can be attended to by well-prepared and protected nurses and doctors. This technological innovation also prevents the transmission of infection among patients who are waiting for their appointment at the clinic. Furthermore, it can limit personal protective equipment use and reduce costs without compromising with the safety of health care workers.

The telepresence robot is a Double model used with the virtual platform developed by the start-up company, Pluginbot. It has a screen on which the patient can see the nurse and a camera through which the nurse can see and interact with the patient from the remote location (Figure 1). It is also mobile – that is, it can be used to guide the patient to a specific room or area, where the patient can obtain a proper face mask or wash his/her hands.

We do not have any mathematical or statistical results on the success of this strategy; however, all professionals involved in patient care or assistance have felt much more confident and secure after the robot service has been initiated. Moreover, patients who arrived at the clinic and made first contact with this robotic system seem to have been more attentive, with improved compliance with the given directions and recommendations. Interestingly, guidance provided by the robot appeared more reliable and dependable from a patient’s perspective.

This is indeed a very interesting technological innovation, especially in the current scenario of COVID-19, a highly contagious disease, wherein we have to put all of our thoughts and ideas into action with the ultimate aim of minimizing virus transmission and fatalities from the disease.

## Figures and Tables

**Figure 1:**
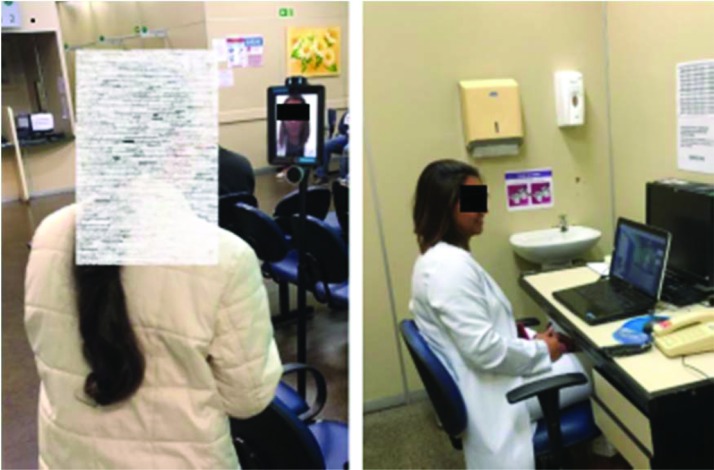
Screening of incoming patients with or without respiratory symptoms using the robot.
